# Polydatin as a natural ClpP modulator for combating methicillin-resistant *Staphylococcus aureus* infection

**DOI:** 10.3389/fcimb.2026.1744317

**Published:** 2026-03-19

**Authors:** Ying Qin, Jingjing Yang, Guangming Wang, Luna Yang, Dacheng Wang, Yongxin Luan

**Affiliations:** 1 Department of Neurosurgery, First Hospital of Jilin University, Changchun, China; 2 College of Animal Science, Jilin University, Changchun, China; 3 Department of Pharmacology, College of Basic Medical Sciences, Jilin University, Changchun, China

**Keywords:** antibiotic resistance, caseinolytic protease P (ClpP), hemolysis inhibition, methicillin-resistant *Staphylococcus aureus* (MRSA), pneumonia model, polydatin

## Abstract

**Introduction:**

Methicillin resistant *Staphylococcus aureus*, MRSA, is a major cause of hospital acquired infections and poses a serious therapeutic challenge because of multidrug resistance and potent virulence. Targeting virulence rather than bacterial growth may provide an alternative strategy to combat MRSA infection. This study investigated polydatin, a stilbenoid glucoside from *Polygonum cuspidatum*, as a potential antivirulence agent targeting caseinolytic protease P, ClpP.

**Methods:**

ClpP inhibitory activity was evaluated by enzymatic assay. The effects of polydatin on bacterial growth, hemolytic activity, virulence gene expression, adhesion to fibrinogen, and host cell invasion were assessed *in vitro*. Target engagement was examined by thermal shift assay, fluorescence quenching, and computational simulation. Therapeutic efficacy was evaluated in a murine pneumonia model.

**Results:**

Polydatin showed limited antibacterial activity but significantly inhibited ClpP and reduced the expression of key virulence factors, including Hla, PVL, and RNAIII. It also impaired bacterial adhesion to fibrinogen and invasion of host cells. Binding studies supported the interaction between polydatin and ClpP. *In vivo*, polydatin markedly alleviated *S. aureus* induced pneumonia, as shown by reduced lung bacterial burden, lower inflammatory cytokine levels, and attenuated tissue injury.

**Discussion:**

Polydatin attenuates MRSA pathogenicity by targeting ClpP associated virulence regulation rather than bacterial viability. These findings identify polydatin as a promising antivirulence candidate and provide a basis for developing alternative therapeutic strategies against MRSA infections.

## Introduction

Methicillin-resistant *Staphylococcus aureus* (MRSA) remains a leading cause of healthcare-associated infections, including pneumonia, skin and soft tissue infections, bloodstream infections, and device-associated infections (Shao et al., 2025). Despite advances in infection control, MRSA continues to pose a major therapeutic challenge due to its mecA-mediated resistance to β-lactam antibiotics and its capacity to establish persistent infections through biofilm formation (McCarthy et al., 2015). Multidrug-resistant phenotypes are increasingly prevalent, with high rates of non-susceptibility to fluoroquinolones and clindamycin, and emerging resistance even to last-line agents such as linezolid (Rønning et al., 2025). This progressive narrowing of effective treatment options underscores the urgent need for novel therapeutic strategies that can circumvent conventional resistance mechanisms while effectively attenuating MRSA pathogenicity.

The pathogenicity of *S. aureus* is attributed to a repertoire of virulence factors, including cytolytic toxins such as hemolysins and leukocidins, immune-modulating proteins, and a variety of extracellular enzymes (Cheung et al., 2021; Di Bella et al., 2025). These factors collectively enable *S. aureus* to evade host immune responses, establish infections, and cause significant tissue damage (Vandenesch et al., 2012). Despite the urgent need for alternative therapeutic approaches, the development of vaccines or immunotherapies against *S. aureus* has been largely unsuccessful, underscoring the necessity of exploring alternative strategies, such as targeting virulence mechanisms (Campos et al., 2022; Nandhini et al., 2022). Suppression of virulence factor production or activity represents an alternative therapeutic strategy. This approach is particularly appealing, as it minimizes the bactericidal pressure typically associated with conventional antibiotics, thereby potentially reducing the likelihood of resistance development.

Caseinolytic protease P (ClpP), a highly conserved serine protease, plays a central role in bacterial homeostasis by degrading misfolded or damaged proteins and, in conjunction with molecular chaperones, large protein complexes (Mabanglo and Houry, 2022). In addition to its housekeeping functions, ClpP influences the pathogenicity of several bacterial species, including *S. aureus*. In *S. aureus*, ClpP contributes to biofilm formation and regulates virulence by modulating the production of key extracellular proteins (Liu et al., 2017). Deletion of the clpP gene results in a significant reduction in the levels of hemolysin (Hla), a critical virulence factor, as well as the downregulation of multiple extracellular virulence-associated proteins involved in infection and immune evasion, including Panton-Valentine leukocidin (PVL, LukSF-PV) and other secreted toxins and exoproteins (Kahne and Darwin, 2021; Luo et al., 2021; Kim et al., 2022; Queraltó et al., 2023).

The transcriptional regulation of *hla* and other virulence factors is intricately linked to the accessory gene regulator (agr) locus, which orchestrates quorum sensing and mediates the expression of agr-dependent extracellular virulence factors. ClpP-deficient *S. aureus* strains exhibit impaired expression of the agr system, resulting in a cascade of attenuated virulence phenotypes (Illigmann et al., 2021; Ju et al., 2021). In addition, ClpP plays a critical role in bacterial stress response pathways, including oxidative stress management, metal ion homeostasis, SOS-mediated DNA damage repair, and heat shock protein regulation (Michel et al., 2006). These observations highlight the multifaceted contributions of ClpP to *S. aureus* physiology and pathogenicity, making it an attractive target for antimicrobial drug development. Studies employing ClpP-deficient strains in murine infection models have shown a marked reduction in bacterial virulence, further validating its potential as a therapeutic target (Park and Ronholm, 2021; Song et al., 2022).

Recognizing the critical role of ClpP in *S. aureus* infections, our research focused on identifying natural products capable of inhibiting ClpP activity. Natural products derived from diverse biological sources are rich reservoirs of bioactive compounds with unique mechanisms of action, making them ideal candidates for drug discovery (Culp et al., 2022; Reinhardt et al., 2022). Through high-throughput screening, we identified polydatin, a stilbenoid glucoside derived from *Polygonum cuspidatum*, as a promising ClpP inhibitor, as it displayed the lowest IC_50_ among the screened compounds. Polydatin has previously been reported to mitigate *S. aureus*-induced mastitis in mice by inhibiting TLR2-mediated activation of the p38 MAPK/NF-κB signaling pathway (Jiang et al., 2017).

The rapid expansion of multidrug-resistant MRSA and the poor performance of conventional antibiotics against persistent infections and biofilms necessitate therapeutic strategies beyond bactericidal killing. Antivirulence intervention, particularly by targeting global regulators of toxin expression, provides a promising alternative. ClpP is a central protease that controls virulence and stress adaptation in *S. aureus* but remains underexplored as a druggable antivirulence target. Here, we aimed to investigate polydatin as a ClpP modulator and to evaluate its ability to suppress MRSA virulence, thereby establishing the feasibility of ClpP-directed antivirulence therapy.

## Materials and methods

### Bacterial strains and culture conditions

Experimental assays were conducted using both methicillin-sensitive *S. aureus* (MSSA) Newman and the MRSA USA300 strain, the latter known for its hypervirulence and clinical relevance. Isolates SA28, SA37, and SA1B3G (Liaoning University of Traditional Chinese Medicine) were included, and their species identities and *mecA* statuses were confirmed via 16S rRNA sequencing and PCR, respectively. A USA300 Δ*clpP* mutant strain, previously constructed and maintained in our laboratory, was also utilized to assess the role of ClpP in the bacterial response, based on prior literature reports (Jing et al., 2021). *Escherichia coli* BL21(DE_3_), used as the host strain for ClpP protein expression, was cultured in Luria–Bertani (LB) broth or on LB agar (Hopebio, Qingdao, China), whereas *S. aureus* strains were propagated in tryptic soy broth (TSB, Hopebio, Qingdao, China) or on tryptic soy agar (TSA, Hopebio, Qingdao, China) under standard aerobic conditions at 37 °C. Antibiotic supplementation was applied as needed: kanamycin (TransGen Biotech, Beijing, China) at 50 μg/mL for *E. coli* was used to maintain plasmid selection or mutant stability. The relevant information on bacterial strains and cells is summarized in **Table 1**.

**Table 1:** Strains used in this study.

Strains	Source
MRSA USA300	This study
ATCC 29213	This study
ATCC33591	This study
SA37	This study
SA28	This study
SA1B3G	This study
Newman	This study
pET28a:: *clpP* -Bl21(DE_3_)	This study
Human lung adenocarcinoma epithelial cells (293T)	This study
Human lung adenocarcinoma epithelial cells (A549)	This study
USA300 Δ*clpP*	This study

ATCC, American Type Culture Collection; MRSA, methicillin-resistant *Staphylococcus aureus.*

### Reagents and compounds

Polydatin (CAS No. 27208-80-6; purity≥99%; Chengdu Pufei De Biotech Co., Ltd.) was dissolved in dimethyl sulfoxide (DMSO) to prepare a 10 mg/mL stock solution, which was stored at -20 °C until use. Before use, the stock solution was further diluted in the corresponding medium/buffer such that the final DMSO concentration in all working solutions was negligible (≤0.1%, v/v).

### Protein expression and purification

The ClpP protein was expressed in *E. coli* BL21 (DE_3_) harboring the pET28a-*clpP* plasmid. Overnight cultures were inoculated into fresh LB broth containing 50 μg/mL kanamycin and grown at 37 °C until the OD_600_ reached ~0.6. Following induction with 0.5 mM isopropyl-β-D-thiogalactopyranoside (IPTG; TransGen Biotech, Beijing, China), the culture was incubated overnight at 20 °C in a shaking incubator (ZWY-2112B, Zhicheng, Shanghai, China) at 160 rpm. The cells were harvested via centrifugation, resuspended in lysis buffer (40 mM Tris, pH 8.5), and lysed via sonication on ice. The lysate was centrifuged at 18,000 × *g* for 1 h at 4 °C, and the supernatant was applied to a His-Trap affinity column (GE Healthcare). The ClpP protein was eluted via a gradient of imidazole, and its purity was assessed via SDS-PAGE.

### Screening for ClpP inhibitors

The intrinsic hydrolytic activity of ClpP was utilized for screening inhibitors. A 96-well black opaque microplate (Corning, USA) was prepared, with each well containing 10 μM ClpP, 64 μg/mL of each of the 40 test compounds (e.g., flavonoids, stilbenoids, and alkaloid derivatives), and 90 μL of ClpP assay buffer (100 mM HEPES, 100 mM NaCl). 10 μM ClpP, 64 μg/mL of each of the 40 test compounds, and 90 μL of ClpP assay buffer (100 mM HEPES, 100 mM NaCl). The final reaction volume was 100 μL per well. The mixture was incubated at room temperature for 1 h. Subsequently, 10 μM Suc-LeuTyr-AMC (Sigma-Aldrich, USA) was added to each well, and the plate was incubated at 32 °C for 30 min. Fluorescence was measured using an Infinite M200 microplate reader (Tecan, Switzerland) with excitation at 360 nm and emission at 465 nm to evaluate ClpP enzymatic inhibition (Zhang et al., 2024).

### Half-maximal inhibitory concentration (IC_50_) determination for ClpP inhibitors

To determine the IC_50_ values of the polydatin against ClpP, serial dilutions of polydatin (0-256 μg/mL) were prepared in ClpP assay buffer (100 mM HEPES, 100 mM NaCl) and added to a 96-well black opaque plate containing 10 μM ClpP in a final volume of 100 μL. The mixtures were incubated at room temperature for 1 h, followed by the addition of 100 μM Suc-LeuTyr-AMC substrate. The plate was incubated at 32 °C for 30 min, and fluorescence was measured at an excitation wavelength of 360 nm and an emission wavelength of 465 nm via an Infinite M200 spectrophotometer. The enzymatic activity was normalized to that of the control wells (no inhibitor), and the IC_50_ values were calculated by fitting the data to a dose–response curve via nonlinear regression analysis (Zhang et al., 2024).

### Minimum inhibitory concentration assays

The MIC assay was conducted to evaluate the antimicrobial activity of polydatin. Overnight bacterial cultures (USA300 or Newman) were grown in Mueller–Hinton broth (MHB) at 37 °C with shaking (220 rpm) until the optical density at 600 nm (OD_600_) reached 0.4–0.6. The cultures were then diluted in cation-adjusted Mueller–Hinton broth (CAMHB) to an OD_600_ of 0.025, corresponding to approximately 5 × 10^5^ CFU/mL as determined by serial dilution and plate counting. Various concentrations of polydatin (0-512 μg/mL) were added to the diluted cultures in a 96-well plate (100 μL per well, with a final DMSO concentration of 0.1%), and each condition was tested in triplicate. The plates were incubated at 37 °C for 24 h, and the lowest concentration of polydatin that visibly inhibited bacterial growth was recorded. Alternatively, the OD_600_ values were measured spectrophotometrically to confirm growth inhibition (Lambert and Pearson, 2000).

### Growth curve analysis

The effect of the compounds on the growth of *S. aureus* USA300 was evaluated by growth curve analysis. Briefly, overnight cultures were diluted 1:100 into fresh TSB and adjusted to an initial OD_600_ of 0.1. WT supplemented with an equivalent volume of DMSO (WT + DMSO), WT treated with polydatin (64 μg/mL), and sterile TSB alone as the medium-only control. For all DMSO-containing groups, the final DMSO concentration was maintained at 0.1% (v/v). Cultures were incubated at 37 °C with shaking, and OD_600_ values were recorded at regular intervals over 24 h using a microplate reader. Growth curves were generated by plotting OD_600_ versus time.

### Cytotoxicity assay

Cell viability was assessed using an MTT assay (Adan et al., 2016). Briefly, A549 cells were seeded into 96-well plates and treated with polydatin at concentrations ranging from 0 to 64 μg/mL for 24 h at 37 °C. After incubation, the culture medium was removed and replaced with 100 μL of MTT solution (0.5 mg/mL, 3-(4,5-dimethylthiazol-2-yl)-2,5-diphenyltetrazolium bromide; Sigma-Aldrich, USA), followed by an additional 4 h incubation at 37 °C. The supernatant was then discarded, and the resulting formazan crystals were dissolved in DMSO (Sigma-Aldrich, USA). Absorbance was measured at 490 nm using an Infinite M200 microplate reader. Cells treated with an equivalent volume of DMSO were used as the vehicle control and were defined as 100% viability.

### Assessment of polydatin-induced effects on erythrocyte membrane stability *in vitro*

To evaluate the impact of polydatin on erythrocyte membrane stability, defibrinated rabbit blood was prepared by stirring freshly collected blood with glass beads to remove fibrin. Erythrocytes were isolated through three washes with phosphate-buffered saline (PBS; pH 7.4; Solarbio, Beijing, China), centrifuged at 3000 rpm for 10 min, and then resuspended to form a uniform suspension. The cells were incubated with polydatin (0–64 μg/mL) at 37 °C for 30 min. PBS (Solarbio, Beijing, China) and 0.1% Triton X-100 (Sigma-Aldrich, USA) were used as negative and positive controls, respectively. Following incubation, the supernatants were collected after centrifugation, and hemolysis was assessed spectrophotometrically at 540 nm using an Infinite M200 microplate reader and expressed as a percentage relative to the Triton X-100 control.

### Drug safety evaluation in the *Galleria mellonella*

To evaluate the *in vivo* safety of polydatin, *G. mellonella* larvae (2.0–2.5 cm in length, approximately 220 mg) with consistent creamy coloration and active motility were selected. Prior to treatment, the larvae were maintained at room temperature and fasted for 24 h to minimize variability. They were then randomly allocated into four groups (n = 10 per group): two treatment groups receiving polydatin at doses of 20 or 40 mg/kg, a vehicle control group (5% DMSO, 20% polyethylene glycol 300 (PEG300), or 75% PBS), and an untreated control group. Each larva was injected with 10 μL of the corresponding solution into the hemocoel via the last left proleg using a sterile 50 μL microsyringe fitted with a 30-gauge needle (9 mm). After injection, larvae were placed in Petri dishes and incubated at 37 °C in the dark. Survival was monitored for 120 h at 24-h intervals. Toxicity-related signs, including melanization, reduced mobility, and abnormal behavior, were recorded throughout the observation period. Larvae were considered dead when no response was observed upon gentle tactile stimulation (Ramarao et al., 2012; Tsai et al., 2016).

### Hemolysis assay

To evaluate the hemolytic activity of *S. aureus* strains (USA300, Newman, SA28, SA37, and SA1B3G), bacteria were cultured in TSB supplemented with polydatin at 0–64 μg/mL, a concentration range selected to cover the dose window used in subsequent *in vitro* activity assays and to avoid growth inhibition–related artifacts. An equivalent volume of DMSO was added to the vehicle control group, with a final DMSO concentration of 0.1% (v/v) across all groups. Cultures were grown to an OD_600_ of 2.5, and culture supernatants were collected by centrifugation (5,500 × g, 4 °C, 3 min) and filtered through 0.22 μm membranes to remove residual bacterial cells. A reaction mixture containing 100 μL of filtered supernatant, 875 μL of PBS, and 25 μL of defibrinated rabbit blood was incubated at 37 °C for 30 min in 1.5 mL microcentrifuge tubes. After incubation, samples were centrifuged, and the absorbance of the supernatants was measured at 600 nm (Qiu et al., 2012). PBS without bacterial supernatant was used as a negative control to determine baseline hemolysis.

### Fibrinogen adhesion assay

*S. aureus* USA300 was cultured overnight, diluted 1:100 in fresh BHI with or without polydatin and grown at 37 °C with shaking until the OD_600_ reached 0.5. Cultures (100 µL) were added to 96-well plates precoated with bovine fibrinogen (20 µg/mL, 4 °C, overnight). After 2 h of incubation at 37 °C, the wells were washed twice with PBS to remove nonadherent cells. Adherent bacteria were fixed with 25% formaldehyde for 30 min, washed, and stained with crystal violet for 20 min. The absorbance at 570 nm was measured to quantify adhesion (Hou et al., 2018).

### Western blot analysis

The USA300 strain of *S. aureus* was exposed to 64 μg/mL polydatin for 12 h. Following treatment, bacterial cells were pelleted and lysed via RAPI lysis buffer (Beyotime, China) to extract intracellular proteins. Protein concentrations of bacterial lysates were determined using a BCA protein assay kit (Beyotime, China), and equal amounts of total protein (20 μg per lane) were loaded for SDS-PAGE. The culture supernatants and bacterial lysates were subjected to SDS-PAGE on 12% polyacrylamide gels, and the proteins were subsequently transferred onto PVDF membranes (Millipore, USA). The membranes were blocked with blocking solution for 2 h before being probed with specific primary antibodies: rabbit polyclonal anti-alpha-toxin antibody (1:5000, Cat No. S7531, Sigma-Aldrich), anti-PVL LukS subunit antibody (1:5000, Cat No. ab190473, Abcam), and anti-ClpP antibody (1:5000). After incubation, HRP-conjugated goat anti-rabbit IgG secondary antibodies (1:2000, Cat. No. SE134, Solarbio, Beijing, China) were used for detection. Protein bands were visualized and quantified via ImageJ software to assess differences in protein expression.

### qPCR analysis

*S. aureus* was cultured in TSB until the optical density at OD_600_ reached approximately 0.3 (1 × 10^8^ CFU/mL) At this stage, polydatin was added to the culture at a final concentration of 64 μg/mL, followed by further incubation for 24 h at 37 °C with shaking. The bacterial cells were harvested for RNA extraction via the MiniBEST Universal RNA Extraction Kit (Cat No. 9767; TaKaRa, Dalian, China). The RNA integrity and purity were verified via agarose gel electrophoresis. cDNA was synthesized via the PrimeScript RT reagent kit (Cat No. RR047Q, TaKaRa), and qPCR was performed on an ABI 7900HT real-time PCR system (Applied Biosystems, USA). The relative expression of the target genes was normalized to that of the reference gene (16S rRNA) via the comparative Ct method. Each experiment was performed in triplicate to ensure reproducibility. The sequences of the qPCR primers used are listed in **Table 2**.

**Table 2:** Oligonucleotide primers used in this study.

Primers		Nucleotide sequence (5’→ 3’)	Tm (°C)	Amplicon size (bp)	Reference
RT-*16 s-rRNA*	Forward	TGATCCTGGCTCAGGATGA	58	142	(Brakstad et al., 1992)
	Reverse	TTCGCTCGACTTGCATGTA	57		
RT-*agrA*	Forward	GCAGTAATTCAGTGTATGTTCA	56	128	This study
	Reverse	TATGGCGATTGACGACAA	56		
RT-*RNAIII*	Forward	AATTAGCAAGTGAGTAACATTTGCTAGT	57	110	This study
	Reverse	GATGTTGTTTACGATAGCTTACATGC	57		
RT-*hla*	Forward	GGTATATGGCAATCAACTT	55	137	This study
	Reverse	CTCGTTCGTATATTACATCTAT	56		
RT-*luks*	Forward	TTGCTGAACCTGTTGGACCA	58	146	This study
	Reverse	TGGGGTGTTAAAGCAAACGA	58		
RT-*spa*	Forward	CAGCAAACCATGCAGATGCTA	57	154	This study
	Reverse	GCTAATGATAATCCACCAAATACAGTTG	57		
RT-*psmα*	Forward	TAAGCTTAATCGAACAATTC	55	123	This study
	Reverse	CCCCTTCAAATA-AGATGTTCATATC	55		

### Urease activity assay

The urease activity of *S. aureus* USA300 was evaluated via a urease agar base (Cat. No. HB4095; Hopebio, Qingdao, China), which incorporates phenol red (Aladdin, China) as a pH-sensitive indicator. In this system, urease-mediated hydrolysis of urea generates ammonia, increasing the pH and triggering a color transition of the medium from yellow to red. To assess the potential inhibitory effect of polydatin, bacterial cultures were inoculated into urease media supplemented with varying concentrations of the compound. All reactions were carried out in sterile test tubes to maintain consistent environmental conditions (Wei et al., 2022).

Following incubation at 37 °C for up to 48 h, colorimetric changes were recorded daily as an initial qualitative readout of urease activity. For quantitative assessment, the absorbance of the culture supernatants was measured across a full spectrum (350–650 nm) via a microplate reader. The optimal absorbance peak for phenol red in this assay system was identified at 560 nm, and subsequent measurements were conducted at this wavelength to ensure accurate detection of urease-dependent pH shifts.

### A549 cell infection and lactate dehydrogenase assay

A549 cells were seeded into 24-well plates at a density of 2 × 10^4^ cells per well and incubated for 12 h to allow cell attachment and stabilization. After incubation, the medium was replaced with fresh RPMI 1640 containing 10% fetal bovine serum. The cells were then infected with *S. aureus* USA300 at a multiplicity of infection (MOI) of 50 in the presence of increasing concentrations of polydatin (0-64 μg/mL). The infection was maintained for 6 h. Cell viability was assessed via calcein AM/propidium iodide (PI) dual staining (Beyotime, China), which distinguishes live (calcein-positive) cells from dead (PI-positive) cells. Fluorescence images were acquired via a fluorescence microscope (Olympus, Japan). Untreated cells were used as a negative control to determine baseline viability. To evaluate cytotoxicity, culture supernatants were collected after 6 h of incubation and analyzed for LDH release via a commercial LDH detection kit (Beyotime, Beijing, China) according to the manufacturer’s instructions. LDH levels in the supernatants were used as a marker of cellular membrane damage and overall cytotoxicity.

### Fluorescence quenching of ClpP by polydatin

ClpP protein (5 μM) was incubated with increasing concentrations of polydatin (0-50 μM) in PBS for 30 min at room temperature in the dark. Fluorescence spectra were recorded using an excitation wavelength of 280 nm and an emission range from 300 to 400 nm. The fluorescence intensity of ClpP alone and in the presence of polydatin was measured, and the quenching effect was determined by comparing the differences in intensity (Papadopoulou et al., 2005).

### Thermal shift assay

The thermal stability of the ClpP protein in the presence of polydatin was assessed via TSA performed on the Bio-Rad iQ5 system (Bio-Rad Laboratories, USA). The reaction mixtures were prepared in a transparent 96-well PCR plate containing ClpP protein (final concentration: 2 μM), TSA buffer (150 mM NaCl, 10 mM HEPES, pH 7.5), polydatin (64 μg/mL), and SYPRO Orange dye (Cat No. S5692, Sigma-Aldrich), which binds to hydrophobic regions exposed during protein unfolding. The plate was subjected to a temperature gradient from 25 °C to 90 °C, increasing at a rate of 1 °C per min, while fluorescence was recorded at an excitation wavelength of 475 nm and an emission wavelength of 590 nm. The midpoint temperature (Tm), at which 50% of the protein was denatured, was calculated to determine the stabilizing effect of polydatin on ClpP (Jing et al., 2021).

### Molecular docking and molecular dynamics simulation

Molecular docking was performed via AutoDock Vina 1.1.2, a widely used docking program that applies a scoring function to predict the most favorable ligand binding conformations. The three-dimensional (3D) structure of *S. aureus* ClpP (PDB ID: 3V5E) was retrieved from the Protein Data Bank (PDB, www.rcsb.org), whereas the structure of polydatin (PubChem CID: 5281718) was obtained from the PubChem database. Before docking, all water molecules and nonessential ligands were removed from the protein structure, and polar hydrogen atoms were added to optimize the docking process. The docking input files were generated via AutoDockTools 1.5.6, which was employed to define the grid box dimensions around the ClpP active site to ensure comprehensive conformational sampling of the ligand. The docking simulations were executed with an exhaustiveness setting of 24 to improve the search efficiency. The docking results were ranked on the basis of binding free energy scores (kcal/mol), and the top-ranked binding poses were selected for further analysis on the basis of their binding affinity and key interactions with ClpP active site residues. The predicted ligand-protein interactions, including hydrogen bonding, hydrophobic interactions, and π-π stacking, were visualized and analyzed to identify critical residues involved in binding. To refine and validate the docking results, molecular dynamics (MD) simulations were performed via the Amber 14 and AmberTools software suites. The generalized AMBER force field (GAFF) was applied to parameterize polydatin, whereas the ff14SB force field was used for ClpP. The top docking pose from AutoDock Vina was selected as the initial conformation for the MD simulations. Postsimulation analysis was performed to evaluate the stability and conformational dynamics of the polydatin-ClpP complex. The key structural parameters analyzed included the root-mean-square deviation (RMSD), root-mean-square fluctuation (RMSF), radius of gyration (Rg), and binding free energy calculations via the Molecular Mechanics/Generalized Born Surface Area (MM/GBSA) approach, among others.

### Pneumonia model experiment

The work has been reported in line with the ARRIVE guidelines 2.0. To investigate the therapeutic effects of polydatin on *S. aureus*-induced pneumonia, a murine infection model was established in which 6-week-old C57BL/6J female mice weighing approximately 20 g were obtained from the Experimental Animal Center of Jilin University. Each experimental group consisted of eight mice. The pneumonia model was developed following established protocols. Briefly, the mice were anesthetized with ether and placed in an upright position to facilitate intranasal administration of an *S. aureus* suspension directly into the lungs. For survival analysis, the mice were infected with 2×10^7^ CFU/30 μL *S. aureus* and treated subcutaneously with 40 mg/kg polydatin at 1 h and 12 h post infection (Jing et al., 2021). Survival rates were monitored daily, and Kaplan-Meier survival curves were plotted to assess the efficacy of polydatin.

For histopathological and bacterial burden assessments, a reduced *S. aureus* dose of 1×10^7^ CFU/30 μL was used to minimize mortality and enable detailed tissue analysis. The mice were treated with polydatin (40 mg/kg) at 1 h and 12 h post infection. After 48 h, the animals were euthanized, and the lungs were harvested. The right lung tissue was homogenized in sterile PBS for CFU enumeration to evaluate the bacterial load. Left lung tissue samples were fixed in 10% formalin for histological analysis. Hematoxylin and eosin (H&E) staining was conducted on paraffin-embedded lung tissue sections to assess histopathological alterations, including inflammatory cell infiltration, alveolar disruption, and tissue injury resulting from infection and therapeutic intervention. To characterize the recruitment of neutrophils within pulmonary tissues further, immunohistochemical staining for Ly6G, a neutrophil-specific surface marker, was performed.

### Measurement of inflammatory cytokines in lung tissue

To evaluate pulmonary inflammation, lung tissues were collected from infected mice after 24 h of polydatin treatment. After euthanasia, the lungs were excised, thoroughly rinsed with ice-cold PBS to remove residual blood, and homogenized in PBS containing 1% protease inhibitor. The homogenates were centrifuged at 4 °C, and the supernatants were stored at −80 °C for subsequent cytokine analysis. The levels of proinflammatory cytokines, including TNF-α, IL-1β, and IL-6, were measured via enzyme-linked immunosorbent assay (ELISA) kits in accordance with the instructions provided by the manufacturer (Beyotime, China).

### Statistical analysis

All the statistical analyses were performed via GraphPad Prism 9 software (GraphPad Software, San Diego, CA). Kaplan-Meier survival curves were generated to assess survival rates, and log-rank tests were conducted to compare differences between treatment groups. Unless otherwise stated, all *in vitro* experiments were independently repeated at least three times (n ≥ 3 biological replicates), with technical replicates included when applicable. Quantitative data were analyzed via two-tailed unpaired Student’s t tests to evaluate the significance of differences between group means. The results are expressed as the means ± standard deviations (SDs), with *p* values reported in the corresponding figure legends. Statistical significance thresholds were defined as follows: ∗∗∗*p* < 0.0001, **p < 0.001, and *p < 0.01.

## Results

### Polydatin as a ClpP inhibitor

**Figure 1:**
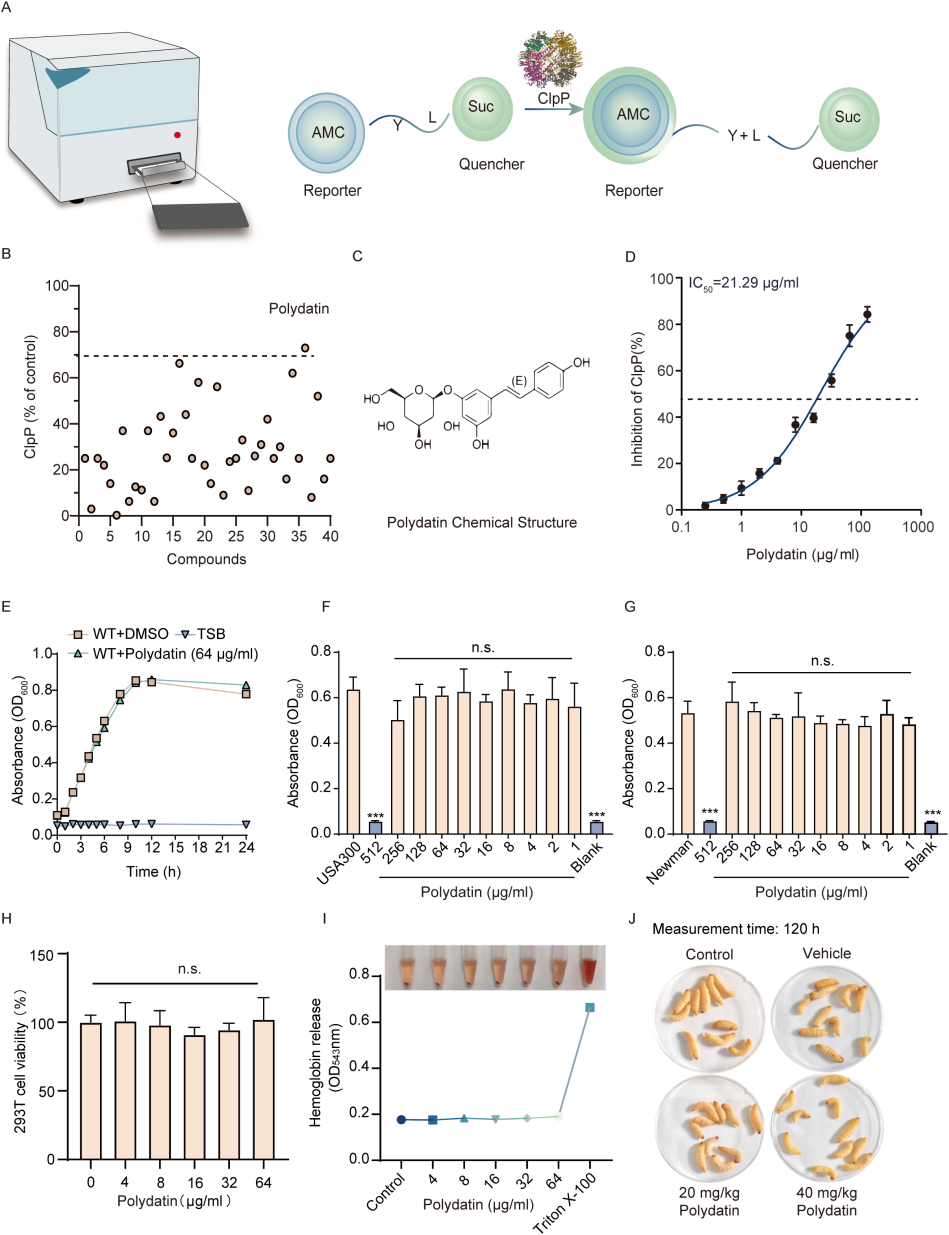
Identification of polydatin as a ClpP inhibitor in *S. aureus*. **(A)** A FRET-based assay was established to screen for ClpP inhibitors, leveraging the ability of ClpP to hydrolyze specific fluorogenic peptide substrates, which emit fluorescence upon cleavage. **(B)** A library of 40 compounds was screened for ClpP inhibition at an initial concentration of 64 μg/mL. The compounds demonstrating >70% inhibition were considered potential candidates. **(C)** Chemical structure of polydatin. **(D)** Dose-dependent inhibition of ClpP activity by polydatin. **(E)** Growth curve analysis showing the effect of polydatin on *S. aureus* USA300 over 24 h. **(F, G)** Determination of the MIC of polydatin against the *S. aureus* USA300 and Newman strains. **(H)** Cytotoxicity assessment of polydatin in mammalian 293T cells. **(I)** Hemolytic activity of polydatin on rabbit erythrocytes across a range of concentrations. **(J)** *In vivo* safety evaluation of polydatin in the *G. mellonella* infection model. For panels with multiple groups **(F–I)**, one-way ANOVA with Tukey’s *post hoc* test was used. n.s., not significant.

ClpP is a proteolytic enzyme responsible for the degradation of misfolded or damaged proteins, and its activity can be accessed via the use of fluorogenic peptide substrates. To identify potential inhibitors of ClpP, we employed a fluorescence resonance energy transfer FRET-based assay, which measures changes in fluorescence intensity upon substrate cleavage, enabling efficient screening of compounds that interfere with ClpP function (**Figure 1A**). Through an initial screen of a 40-compound natural product library (including representative polyphenols and flavonoids), polydatin was identified as a candidate ClpP inhibitor (**Figures 1B, C**), showing a marked inhibitory effect at 64 μg/mL, with a greater than 70% reduction in protease activity (Sassetti et al., 2019). The half-maximal inhibitory concentration (IC_50_) of polydatin was determined to be 21.29 μg/mL (**Figure 1D**). To assess whether polydatin influences bacterial viability, *S. aureus* USA300 was cultured in the presence of 64 μg/mL polydatin. No significant alteration in bacterial growth kinetics was observed (**Figure 1E**), indicating that polydatin does not exert growth-inhibitory effects at concentrations effective for ClpP inhibition. Additionally, the MIC of polydatin against both MRSA USA300 and MSSA Newman strains was 512 μg/mL (**Figures 1F, G**), confirming its lack of direct antimicrobial activity. Together, these results indicate that polydatin selectively targets ClpP protease activity without compromising bacterial growth, suggesting a low risk of inducing selective pressure or promoting the development of antimicrobial resistance in *S. aureus* populations.

### Safety profile of polydatin

The safety profile of polydatin was evaluated through a series of *in vitro* and *in vivo* experiments. *In vitro* cytotoxicity assays were performed on mammalian cell lines at concentrations ranging from 0 to 64 μg/mL, revealing no significant reduction in cell viability, indicating low toxicity (**Figure 1H**). Hemolytic activity was also assessed by incubating polydatin with defibrinated rabbit red blood cells (RBCs) at the same concentrations, and no hemolysis was observed, confirming that polydatin does not compromise RBC integrity (**Figure 1I**). To assess *in vivo* toxicity, the *G. mellonella* model was employed, where larvae were treated with polydatin at doses of 20 mg/kg and 40 mg/kg. No signs of melanization, a common marker of toxicity, were observed, and the larvae remained healthy, further indicating that polydatin does not cause acute systemic toxicity (**Figure 1J**). These findings collectively suggest that polydatin possesses a favorable safety profile, demonstrating low cytotoxicity, an absence of hemolytic activity, and no observable *in vivo* toxicity, suggesting that it is a promising candidate for therapeutic use.

### Polydatin inhibits hemolysis, PVL production, and bacterial adhesion

**Figure 2:**
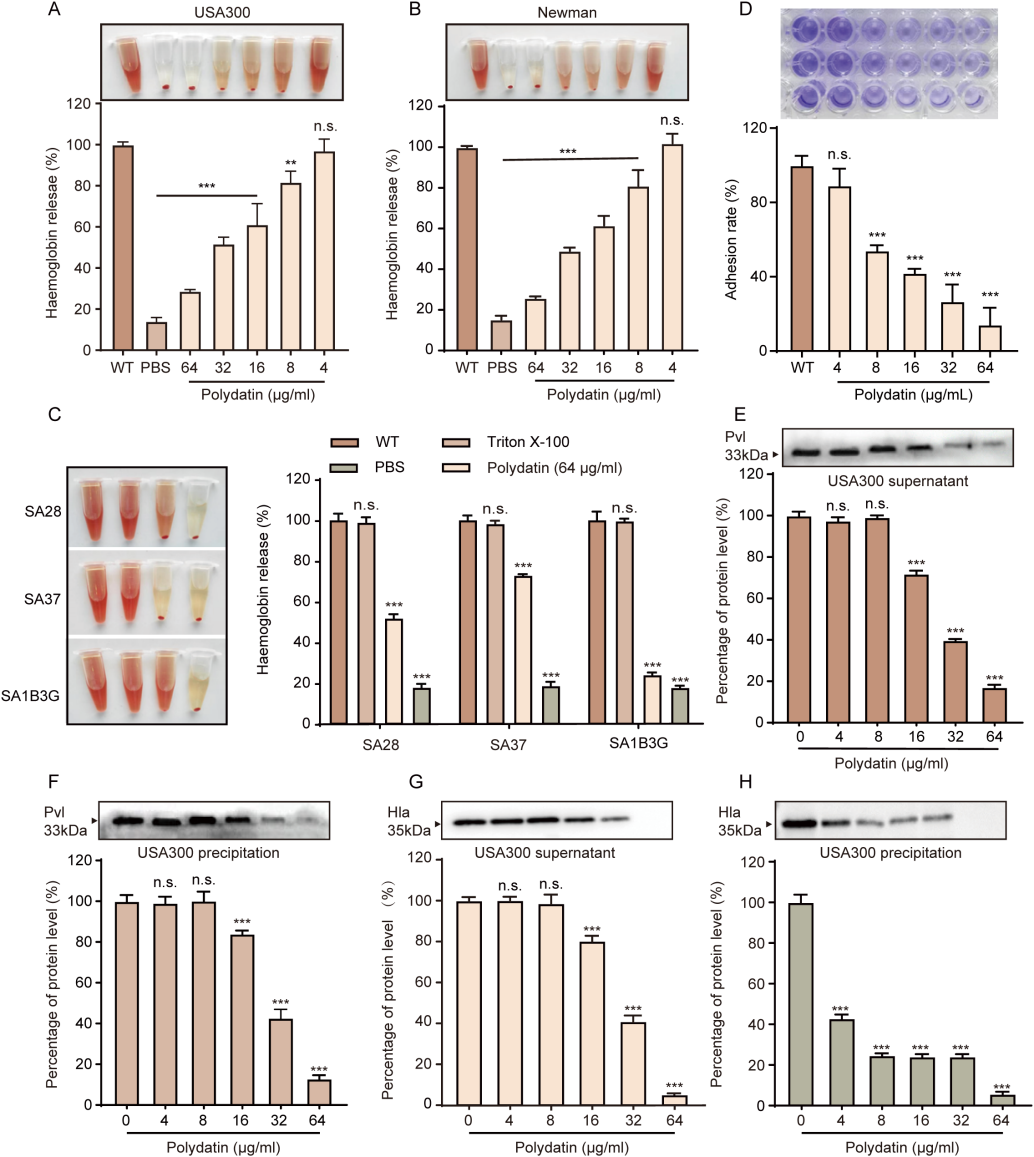
Polydatin inhibits hemolysis, fibrinogen adhesion, and the expression of hla and PVL. **(A, B)** Polydatin inhibited the hemolytic activity of the *S. aureus* USA300 and Newman strains in a dose-dependent manner. **(C)** Polydatin (64 μg/ml) significantly reduced hemolysis in the clinical isolates SA28, SA37, and SA1B3G. **(D)** Dose-dependent inhibition of fibrinogen-mediated adhesion of *S. aureus* by polydatin. **(E, F)** Western blot analysis revealed that polydatin suppressed the expression of PVL in both bacterial pellets and culture supernatants of USA300 in a dose-dependent manner. **(G, H)** Western blot analysis revealed that polydatin suppressed the expression of Hla in both bacterial pellets and culture supernatants of USA300 in a dose-dependent manner. One-way ANOVA with Dunnett’s *post hoc* test (vs WT) was used for multiple-group comparisons. n.s., not significant; **P < 0.01, ***P < 0.001.

To investigate the impact of polydatin on the virulence of *S. aureus*, we first assessed its effect on hemolytic activity. Polydatin (64 μg/mL) significantly inhibited the hemolysis induced by the culture supernatants of the *S. aureus* USA300 and Newman strains in a dose-dependent manner, as evidenced by the reduced lysis of rabbit red blood cells (**Figures 2A, B**). Similar inhibitory effects were also observed across a panel of clinical *S. aureus* isolates, indicating a consistent attenuation of hemolytic capacity across multiple strains (**Figure 2C**). In addition to hemolysis, polydatin markedly suppressed the adhesion of *S. aureus* to fibrinogen-coated surfaces. At a concentration of 64 μg/mL, bacterial adherence was reduced to approximately one-fifth that of the control group, suggesting that polydatin interferes with surface adhesion mechanisms critical for colonization and biofilm formation (**Figure 2D**). PVL, a bicomponent pore-forming cytotoxin secreted by *S. aureus*, plays a pivotal role in immune evasion by targeting and lysing neutrophils and other immune cells. Quantitative analysis revealed that polydatin significantly suppressed PVL production in both the USA300 and Newman strains in a concentration-dependent manner (**Figures 2E, F**), indicating its potential to impair the cytotoxicity of *S. aureus* toward host immune defenses. Furthermore, Hla, a key virulence factor involved in host cell lysis and tissue destruction, was substantially downregulated following polydatin treatment. Both the extracellular (supernatant) and the cell-associated (pellet) levels of Hla protein were reduced by more than fourfold in MRSA USA300 cultures exposed to polydatin, highlighting its strong inhibitory effect on toxin expression (**Figures 2G, H**). Collectively, these findings demonstrate that Polydatin impairs multiple virulence determinants of *S. aureus*, including hemolytic activity, cytotoxin secretion, and host matrix adhesion, without exerting bactericidal effects. This antivirulence approach may provide therapeutic benefits by disarming pathogenic bacteria while minimizing selective pressure for resistance development.

### Effect of polydatin on the transcriptional regulation of virulence genes in *S. aureus* USA300

**Figure 3:**
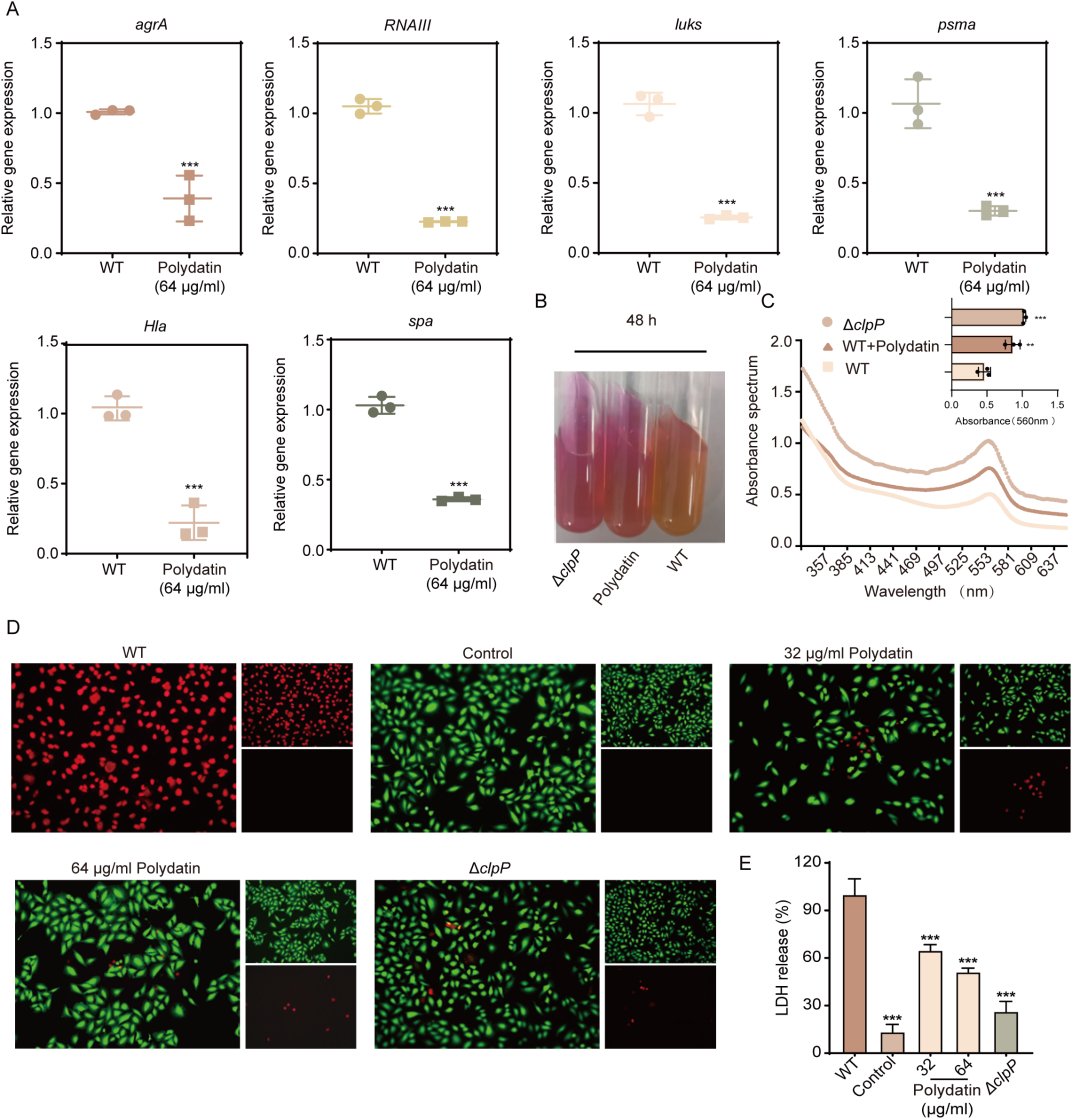
Polydatin attenuates *S. aureus* virulence and protects host cells from MRSA-induced damage. **(A)** qPCR analysis showing the effect of polydatin on the transcription levels of *S. aureus* virulence-related genes, including *agrA*, *RNAIII*, *lukS*, *psmα*, *hla*, and *spa*. **(B)** Urease activity assay assessing the effect of polydatin on the production of urease by *S. aureus*. **(C)** Absorbance spectra of treated samples were recorded across the full wavelength range, with specific measurements at 560 nm used to quantify changes in urease activity. **(D)** Live/dead staining of A549 epithelial cells following infection with *S. aureus* and treatment with polydatin. Green fluorescence indicates viable cells, whereas red fluorescence indicates dead cells. **(E)** LDH release assay was performed on the supernatants of infected A549 cells to assess cytotoxicity, demonstrating the protective effect of polydatin. Comparisons between two groups were conducted using two-tailed Student’s *t*-test, and multiple-group comparisons were analyzed by one-way ANOVA followed by Dunnett’s *post hoc* test (vs WT). A *P* value < 0.05 was considered statistically significant; n.s., not significant; ***P < 0.001.

ClpP, a central protease in *S. aureus*, plays a pivotal role in modulating virulence by influencing the expression of numerous pathogenic determinants. To elucidate the regulatory impact of polydatin on *S. aureus* virulence at the transcriptional level, we conducted quantitative reverse transcription PCR (qRT-PCR) with a panel of key virulence-associated genes in the USA300 strain. Polydatin (64 μg/mL) treatment led to significant repression of *agrA* and *RNAIII*, the core components of the accessory gene regulator (*agr*) quorum-sensing system. As global regulators, these genes orchestrate the expression of a broad array of virulence factors essential for the transition from colonization to invasive infection. Suppression of the agr system consequently resulted in reduced transcription of several downstream effectors. Notably, polydatin significantly downregulated *spa*, which encodes protein A, a major surface adhesin involved in immune evasion and epithelial adherence. In parallel, the transcriptional levels of critical pore-forming cytotoxins including *hla*, *lukS*, and *pmsα* were markedly diminished following treatment (**Figure 3A**). In addition, urease plays a pivotal role in the acidic stress response of *S. aureus*, contributing not only to its adaptation under hostile conditions but also to its virulence. In this study, we explored the impact of polydatin on urease activity in *S. aureus* via the use of a specialized urease agar medium. Notably, both the ClpP-deficient strains and those treated with polydatin presented a distinct red color change in the medium, which was indicative of increased urease production (**Figures 3B, C**). These results suggest that polydatin induces the upregulation of urease, which consequently disrupts the intracellular pH balance of bacteria. Moreover, these findings imply that this effect may be mediated through the ClpP protease, a key regulator of protein quality control and stress responses in *S. aureus*.

### Polydatin enhances host cell survival by increasing *S. aureus*-induced cytotoxicity

Adherence and host cell survival are critical determinants during the early phase of *S. aureus* infection. In this study, polydatin significantly attenuated *S. aureus*-induced cytotoxicity in A549 human lung epithelial cells. Calcein-AM/propidium iodide dual staining revealed a marked, dose-dependent increase in cell viability following polydatin treatment, with notably fewer dead cells than in the untreated control. At 64 µg/mL, polydatin preserved cell integrity to a substantial degree, highlighting its protective role against bacterial insult (**Figure 3D**). To further quantify the cytoprotective effects, LDH release was measured as an indicator of cell membrane integrity. Consistent with the results of the fluorescence-based viability assay, polydatin markedly reduced LDH release from infected cells, suggesting effective prevention of *S. aureus*-induced lytic damage (**Figure 3E**). Together, these findings suggest that polydatin not only disrupts bacterial adherence but also confers robust protection to host epithelial cells during infection.

### Polydatin interacts with ClpP and modulates its stability

**Figure 4:**
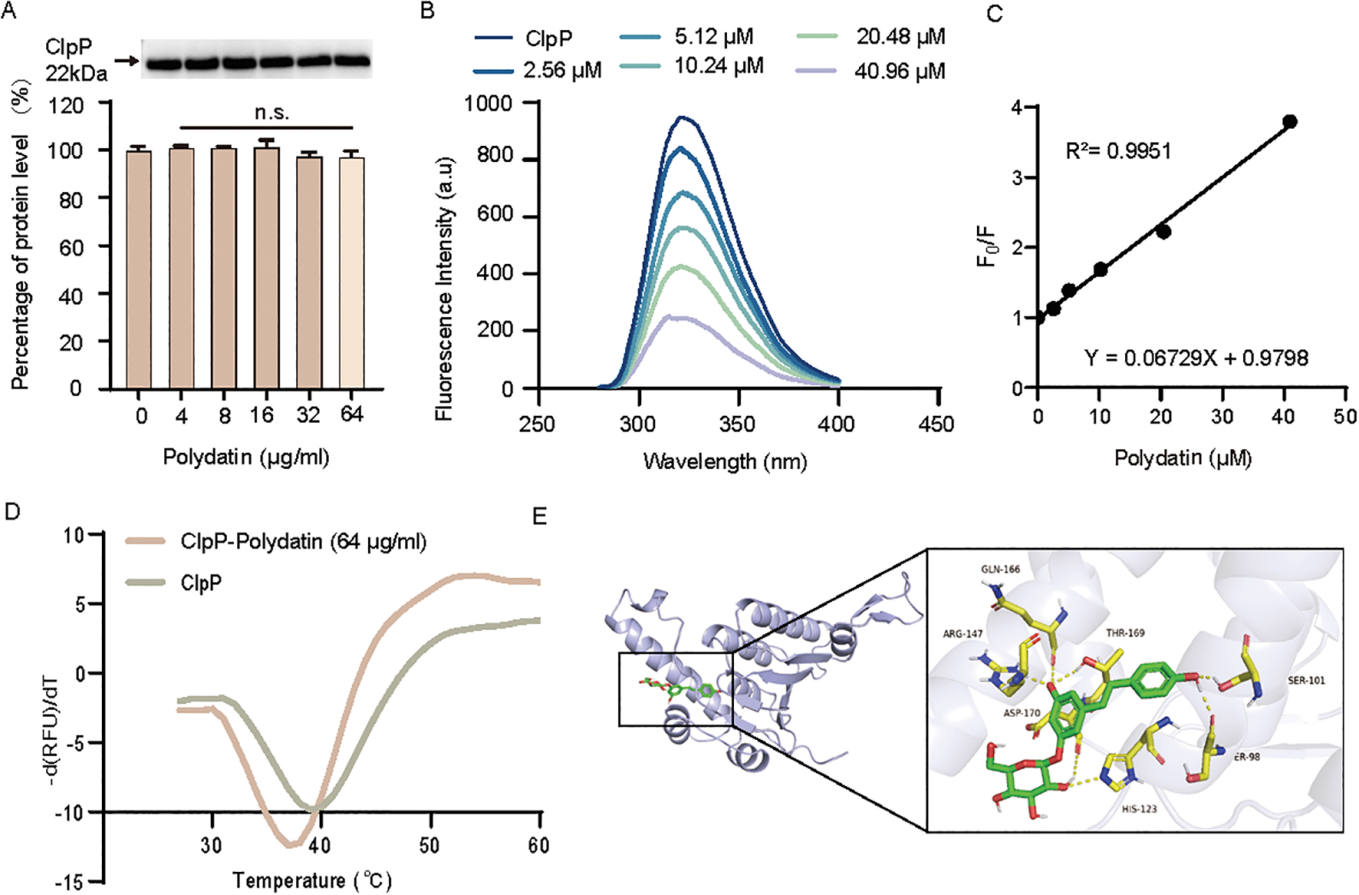
Polydatin directly targets ClpP. **(A)** Western blot analysis showing the effect of Polydatin on ClpP protein expression. **(B)** Fluorescence quenching analysis indicating a direct interaction between Polydatin and ClpP, with a calculated binding constant (K_A_) of 5.95 × 10^4^ L/mol. The relationship between F_0_/F and the polydatin concentration was plotted to assess the binding affinity, where F_0_ represents the fluorescence intensity of ClpP in the absence of Polydatin, and F represents the fluorescence intensity in its presence. **(D)** TSA curve of the ClpP-Polydatin complex, demonstrating a noticeable shift in the Tm, suggesting altered protein stability upon binding. **(E)** Molecular docking analysis predicting key amino acid residues involved in the interaction between ClpP and Polydatin. For **(A)**, one-way ANOVA followed by Dunnett’s *post hoc* test (vs untreated control) was used. n.s., not significant.

To determine whether polydatin affects the expression of ClpP in *S. aureus*, we first conducted Western blot analysis. The results revealed no significant change in ClpP protein levels upon polydatin treatment, indicating that polydatin does not regulate ClpP expression at the protein level (**Figure 4A**). To evaluate the direct molecular interactions between polydatin and ClpP, we next performed intrinsic fluorescence quenching assays. ClpP exhibits native fluorescence due to aromatic amino acid residues, with a characteristic emission peak at approximately 280 nm. Upon the addition of polydatin, a marked, concentration-dependent reduction in fluorescence intensity was observed, suggesting that a direct interaction between polydatin and ClpP alters the local microenvironment. The binding constant (*K_A_*) was calculated to be 5.95 × 10^4^ L/mol, further supporting the existence of a specific binding interaction (**Figures 4B, C**). Given the importance of protein–ligand binding in elucidating the molecular mechanisms of action, we employed a TSA to assess the impact of polydatin on ClpP stability. TSA measures alterations in the thermal denaturation profile of a protein upon ligand binding. As shown in **Figure 4D**, the Tm of ClpP increased by approximately 2 °C following polydatin treatment, indicating enhanced thermal stability and suggesting that polydatin binding stabilizes the ClpP structure. To further explore the binding interface, molecular docking simulations were performed to predict the interaction mode between polydatin and ClpP. The calculated binding free energy was −8.2 kJ/mol, which is consistent with favorable and energetically stable interactions (**Figure 4E**). Collectively, these findings provide strong evidence that polydatin directly binds to ClpP, altering its conformational stability and potentially modulating its proteolytic function.

### Structural stability and interaction dynamics of the ClpP-polydatin complex revealed by molecular simulations

**Figure 5:**
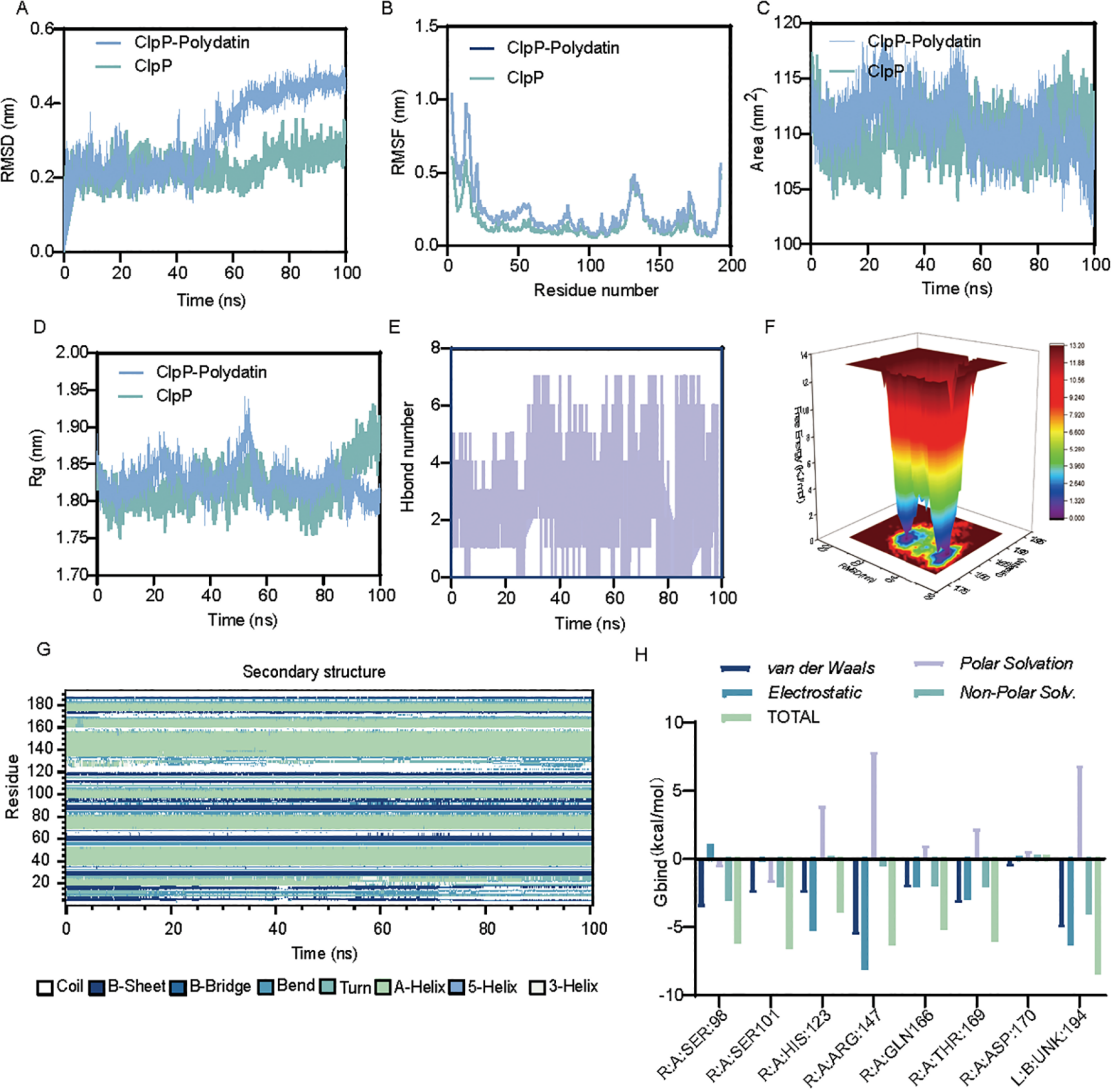
Structural stability and interaction dynamics of the ClpP-polydatin complex revealed by molecular simulations. **(A)** RMSD profiles indicating the structural stability of both systems throughout the simulation. **(B)** RMSF analysis showing reduced flexibility in the N-terminal region of ClpP upon polydatin binding. **(C)** SASA trends revealing a slightly more compact conformation in the ClpP-polydatin complex. **(D)** Radius of gyration (Rg) values confirming overall structural compactness were maintained. **(E)** Time evolution of hydrogen bonds between ClpP and polydatin. **(F)** Potential energy surface (PES) highlighting energetically favorable binding conformations. **(G)** Secondary structure analysis showing that α-helices and β-sheets remained conserved during binding. **(H)** Per-residue free energy decomposition identifying key residues contributing to ligand binding through van der Waals and electrostatic interactions.

On the basis of the molecular docking results, MD simulations were performed to further evaluate the stability and interaction profile of the ClpP-polydatin complex. RMSD analysis indicated that the protein structures of both systems remained stable throughout the simulation period (**Figure 5A**). The RMSF profiles were compared to assess residue-level flexibility. Both apo ClpP and the ClpP–polydatin complex displayed similar overall fluctuation patterns, with greater flexibility observed at the N-terminal region (residues 1-40) and the C-terminal tail. Notably, residues in the ClpP–polydatin complex exhibited slightly reduced fluctuations, particularly within the N-terminal domain, suggesting that polydatin binding enhances local structural stability (**Figure 5B**). Time-dependent solvent-accessible surface area (SASA) analysis revealed that the ClpP-polydatin complex maintained a slightly lower and more stable SASA than unbound ClpP did, implying a more compact and stabilized conformation upon ligand binding (**Figure 5C**). Consistently, the radius of gyration (Rg) values remained relatively stable in both systems, with only minor fluctuations, indicating that polydatin does not disrupt the overall compactness or structural integrity of ClpP (**Figure 5D**). Hydrogen bond analysis revealed that an average of 2–6 stable hydrogen bonds were maintained between ClpP and polydatin during the 100 ns simulation, suggesting persistent and favorable interactions (**Figure 5E**). The potential energy surface (PES) of the complex exhibited distinct minima, indicating energetically favorable binding conformations and further supporting the stability of the interaction (**Figure 5F**). Secondary structure analysis revealed that the overall secondary elements of ClpP, including α-helices and β-sheets, were well preserved in the presence of polydatin, indicating that no significant conformational disruption occurred upon ligand binding (**Figure 5G**). Finally, residue binding free energy decomposition identified several key residues, such as HIS123, ARG147, and GLN166 that contributed substantially to ligand binding, primarily through van der Waals and electrostatic interactions. These residues are likely essential for stabilizing the ClpP-polydatin complex (**Figure 5H**).

### Polydatin confers protection against lethal MRSA pneumonia in mice

**Figure 6:**
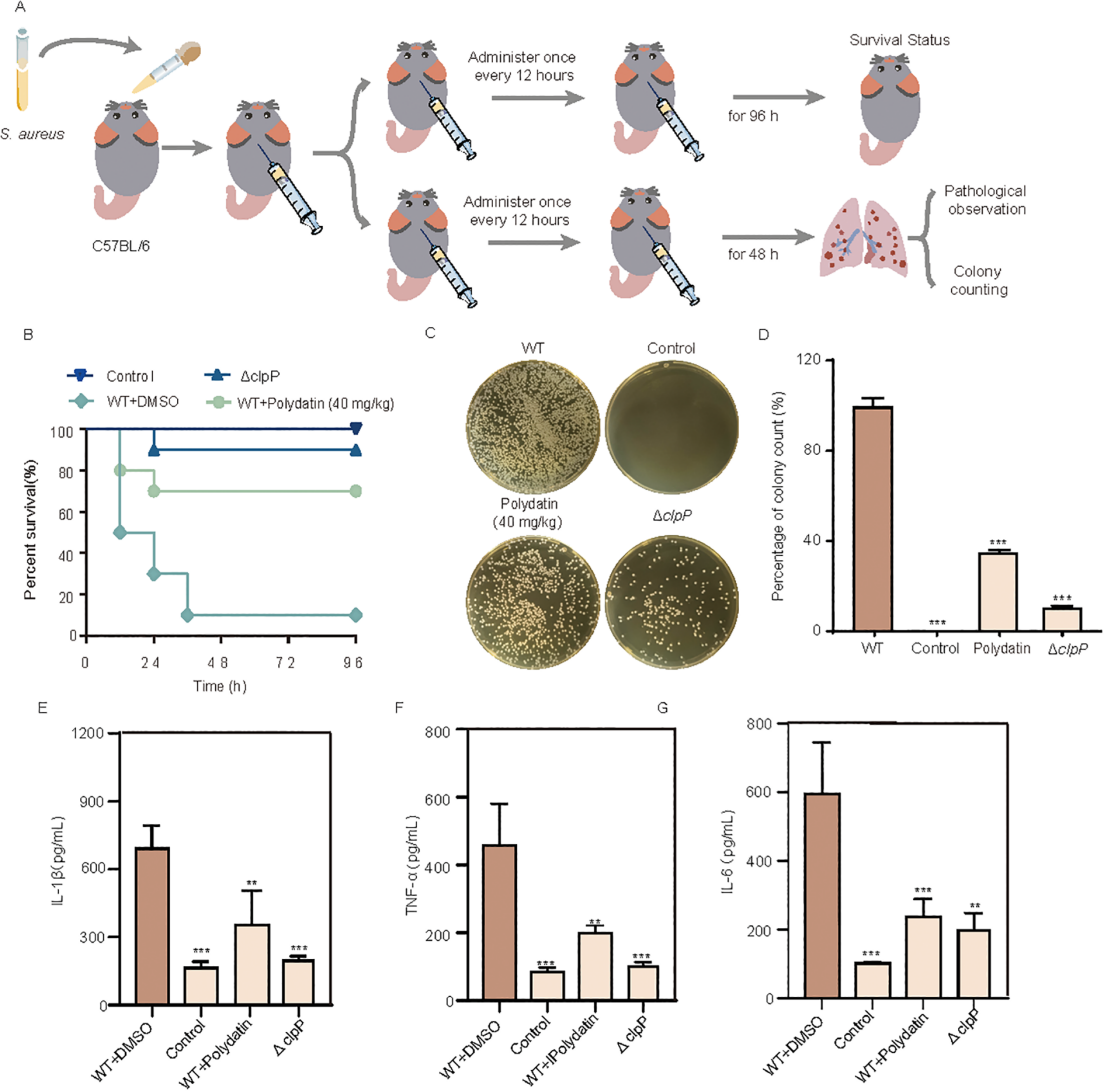
Polydatin improves survival and reduces bacterial burden and inflammation in a murine model of *S. aureus* infection. **(A)** Schematic of the murine infection model. C57BL/6 mice were infected with *S. aureus* and treated with polydatin (40 mg/kg, subcutaneously, every 12 h). Survival was monitored for 96 h, and lung tissues were collected at 48 h for bacterial load quantification and pathological assessment. **(B)** Kaplan-Meier survival curves showing that compared with vehicle treatment, polydatin treatment significantly improved survival in infected mice. **(C)** Representative images of bacterial colony formation from lung homogenates across different groups. **(D)** Quantification of the bacterial burden. **(E-G)** The levels of the proinflammatory cytokines IL-1β, TNF-α, and IL-6 in lung tissue homogenates were markedly lower in the polydatin-treated and Δ*clpP* groups than in the infected controls. Survival curves were analyzed by Kaplan–Meier with log-rank test. CFU counts and cytokine levels were analyzed by one-way ANOVA with Dunnett’s *post hoc* test (vs WT + DMSO). *P* < 0.05 was considered significant. **P < 0.01, ***P < 0.001.

**Figure 7:**
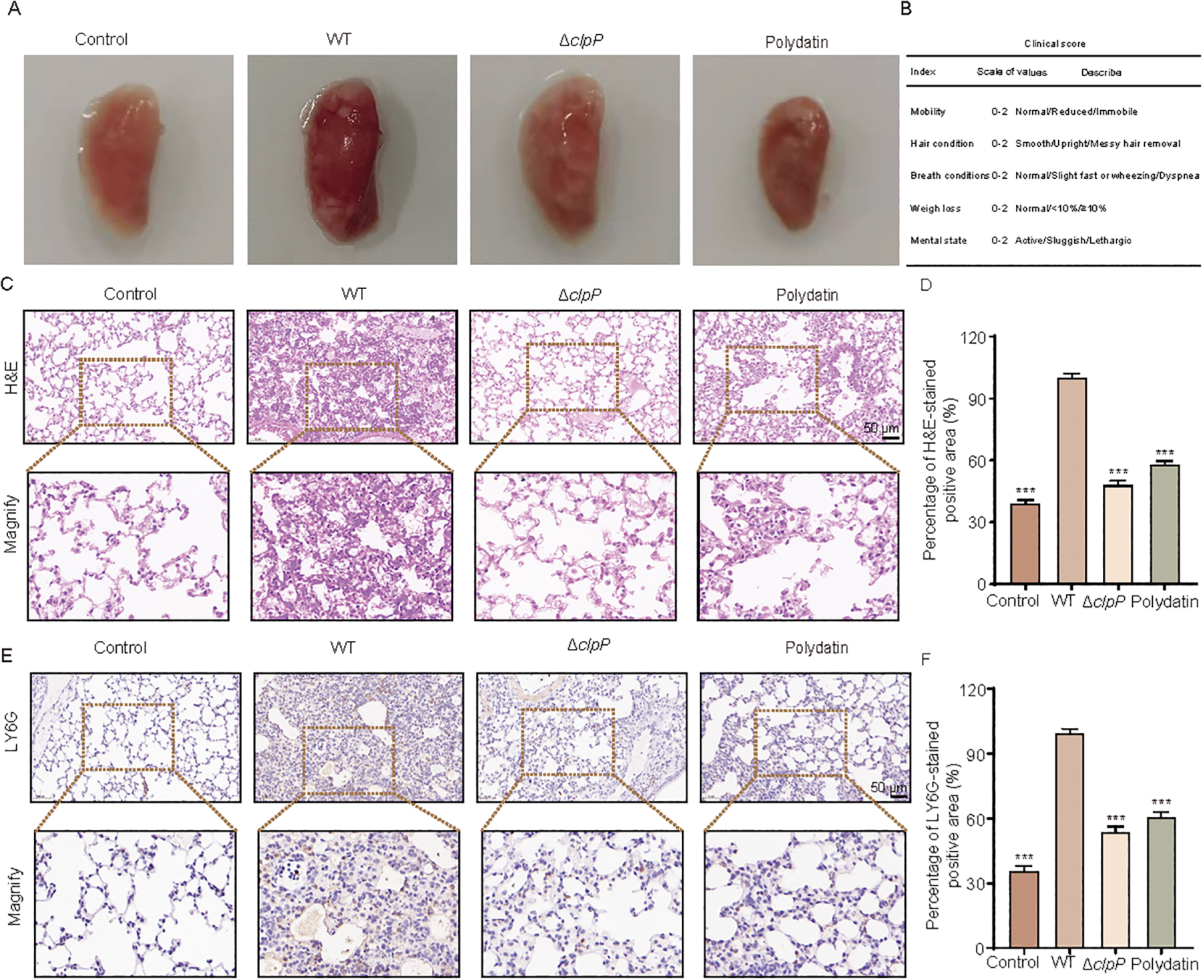
Polydatin attenuates lung damage and neutrophil infiltration in *S. aureus*-infected mice. **(A)** Gross morphology of lungs from each group, showing severe congestion and consolidation in the WT-infected mice, which were alleviated in the Δ*clpP* and polydatin-treated groups. **(B)** Clinical scoring criteria based on mobility, fur condition, respiratory signs, weight loss, and mental state. **(C)** Representative H&E-stained lung sections. The WT group displayed extensive alveolar destruction and inflammatory infiltration, which were markedly reduced in the Δ*clpP* and polydatin groups. **(D)** Quantification of H&E-stained inflammatory areas revealed a significant reduction in lung injury following Δ*clpP* deletion or polydatin treatment. **(E)** Immunohistochemical staining for Ly6G, a neutrophil marker, revealed extensive neutrophil infiltration in the WT group, which was substantially decreased in the Δ*clpP* and polydatin groups. **(F)** Quantification of the Ly6G-positive area revealed significantly reduced neutrophil accumulation in both the Δ*clpP* and polydatin-treated mice. One-way ANOVA followed by Dunnett’s *post hoc* test (vs WT group) was used. n.s., not significant; ***P < 0.001.

To evaluate the therapeutic efficacy of polydatin in MRSA-induced pneumonia, mice were intranasally infected with either the wild-type USA300 strain or a *clpP*-deficient mutant (USA300-Δ*clpP*), followed by the administration of polydatin. Survival was monitored over a 96-h period (**Figure 6A**). As shown in **Figure 6B**, mice infected with wild-type USA300 exhibited a survival rate of only 10%. In contrast, infection with USA300-Δ*clpP* led to a markedly improved survival rate of 90%, underscoring the importance of ClpP in MRSA virulence. Notably, treatment with polydatin at a dose of 40 mg/kg significantly increased the survival of USA300-infected mice, with a survival rate of 70% at 96 h. These findings highlight the potent protective effect of polydatin in a lethal model of MRSA pneumonia. To further elucidate the protective mechanisms of polydatin, the bacterial burden in the lungs was quantified 48 h post infection. Compared with untreated controls, mice treated with polydatin presented a significant reduction in the lung bacterial load, as reflected by lower CFU counts in the lung homogenates, indicating effective suppression of bacterial proliferation *in vivo* (**Figures 6C, D**). We next assessed the pulmonary inflammatory response by measuring the levels of key proinflammatory cytokines in lung tissues. The ELISA results revealed that Polydatin treatment markedly decreased the TNF-α, IL-1β, and IL-6 concentrations in the mice infected with wild-type USA300, suggesting that polydatin alleviates infection-induced inflammation and may promote a more controlled immune response (**Figures 6E–G**). Gross examination of the lung tissues further demonstrated the protective effects of polydatin. Lungs from infected, untreated mice appeared dark red, firm, and consolidated, with visible signs of hemorrhage and edema-hallmarks of severe inflammation and tissue damage. In contrast, lungs from polydatin-treated mice presented a markedly improved appearance, characterized by a lighter color, reduced consolidation, and a more elastic, spongy texture (**Figure 7A**). Furthermore, clinical scores were assigned to each group according to the criteria shown in **Figure 7B**. Compared with those in the untreated infected group, the outcomes of the Polydatin-treated group were significantly improved, with notably higher clinical scores following treatment. Additionally, improvements in respiratory conditions, fur appearance, and overall mental state were observed, further supporting the therapeutic efficacy of polydatin. Histopathological evaluation further corroborated these findings. H&E staining of lung sections from infected, untreated mice revealed severe alveolar damage, extensive pulmonary congestion, interstitial edema, and dense inflammatory infiltrates. In contrast, lungs from polydatin-treated mice presented significantly ameliorated pathology, with reduced alveolar wall thickening and diminished cellular infiltration, closely resembling those of the uninfected control group (**Figures 7C, D**).

To assess neutrophil recruitment, we performed immunohistochemical staining for Ly6G, a neutrophil surface marker. Lung tissues from untreated infected mice exhibited intense Ly6G-positive staining, indicating massive neutrophil infiltration. Compared with control mice, polydatin-treated mice presented markedly fewer Ly6G-positive cells within the alveolar and interstitial spaces, reflecting attenuation of neutrophilic inflammation (**Figures 7E, F**). This reduction in neutrophil infiltration further supports the anti-inflammatory role of polydatin during MRSA-induced lung infection. Collectively, these results demonstrate that polydatin confers significant protection in a murine model of MRSA pneumonia by reducing the bacterial burden, modulating host inflammatory responses, and preserving the lung tissue architecture. These findings position polydatin as a promising antivirulence therapeutic candidate for the management of severe *S. aureus* infections.

## Discussion

The rate of antibiotic resistance development currently exceeds the rate at which new drugs are developed. This threatens to put an end to the golden age of antibiotics and leads to the start of the postantibiotic era. This would have a far-reaching effect on medical procedures, as they all rely on the use of antibiotics to manage infections (Abbas et al., 2024; Yang and Grossart, 2024).

Plants are an enormous treasure trove of antibacterial molecules and sensitizers against MRSA. Plants contain a wide variety of secondary metabolites that can resist external stress and pathogenic attacks. These compounds are classified into terpenoids, alkaloids, flavonoids, polyphenols, coumarins, and fatty acids (Shin et al., 2018). The advent of high-throughput screening methods for the assessment of large amounts of plant extracts containing putative biologically active compounds has encouraged industrial interest in plant research. Numerous studies have reported that plant-derived small molecules increase the sensitivity of MRSA to antibiotics. This could constitute a new strategy for overcoming MRSA infections (Lade and Kim, 2021).

The cell wall, cell membrane, and virulence factors of *S. aureus* are critical for its survival and pathogenicity. The cell wall is primarily composed of a thick peptidoglycan layer that not only maintains the structural integrity of the cell but also protects it from osmotic stress. The cell membrane, a phospholipid bilayer, serves as a selectively permeable barrier that prevents the entry of harmful substances, including antibiotics, thereby contributing to the resistance mechanisms of bacteria. Virulence factors, which are molecules produced by *S. aureus*, play pivotal roles in the ability of bacteria to establish infections, evade host immune responses, and damage host tissues (Gersch et al., 2013; Nikolic and Mudgil, 2023). These factors include enzymes, toxins, and surface proteins that facilitate adherence to host tissues, promote immune evasion, and enable tissue destruction. Given their essential role in pathogenesis, targeting these virulence factors represents a promising strategy for combating bacterial infections, particularly in a way that avoids the development of antibiotic resistance (Brötz-Oesterhelt and Sass, 2014; Bhandari et al., 2018).

One key regulator of virulence in *S. aureus*, ClpP, a highly conserved protease that is modulated by ATPases in both bacteria and human mitochondria. ClpP plays a crucial role in protein quality control by facilitating the degradation of misfolded or damaged proteins. In bacteria, ClpP is involved in regulating the expression of virulence factors, antibiotic resistance, and biofilm formation, as well as the persistence of bacteria under stress conditions. By controlling these processes, ClpP significantly influences the pathogenic potential of *S. aureus* and its ability to survive in hostile environments, such as during antibiotic treatment or in the presence of immune responses.

Our research focused on polydatin, a natural stilbenoid compound derived from the roots of *P. cuspidatum*, which is recognized for its wide therapeutic applications. polydatin has demonstrated significant efficacy in treating a variety of diseases, including lipopolysaccharide (LPS)-induced acute lung injury (ALI) in rat models (Li et al., 2014). Moreover, its well-established anti-inflammatory properties, as evidenced in RAW 264.7 macrophages, highlight its potential for modulating the immune response (Lou et al., 2015). These attributes make polydatin a promising candidate for broader therapeutic interventions, particularly in the context of bacterial infections.

Recent studies have underscored the potential of targeting ClpP as a novel approach to attenuate bacterial virulence and overcome challenges associated with antibiotic resistance (Gronauer et al., 2024). In this context, our findings suggest that polydatin exerts a modulatory effect on ClpP activity, leading to the downregulation of critical *S. aureus* virulence factors. Polydatin inhibits the proteolytic function of ClpP, thereby reducing the expression of PVL and Hla, which are pivotal in the ability of bacteria to evade host immune responses and cause damage. Importantly, the reduction in PVL and Hla protein levels observed by Western blotting was consistent with the decreased transcription of their corresponding genes detected by qRT-PCR, indicating that polydatin suppresses these virulence determinants at both transcriptional and translational levels. The suppression of these virulence factors highlights the therapeutic potential of polydatin in mitigating the pathogenicity of *S. aureus*, especially in contexts where traditional antibiotics may fail due to resistance.

Given that polydatin also impairs *S. aureus* adhesion to fibrinogen and mitigates damage to A549 epithelial cells, we further extended our investigation to evaluate its therapeutic potential in a murine model of *S. aureus*-induced pneumonia. The ability of polydatin to decrease bacterial adhesion to host tissues and its subsequent effects on invasion support the idea that polydatin may be an effective adjunct agent for preventing or treating *S. aureus*-mediated pulmonary infections. By targeting the virulence factors involved in bacterial adherence and immune evasion, polydatin may serve as a complementary therapeutic approach for managing respiratory infections, particularly in the face of increasing antibiotic resistance.

To establish a direct link between polydatin and ClpP, we employed a suite of molecular techniques. FRET assays revealed a potential functional interaction, which was further substantiated by fluorescence quenching analysis and TSAs, both of which provided additional biophysical evidence supporting the binding between polydatin and ClpP. Furthermore, to explore the underlying molecular dynamics, we performed computational kinetic simulations, which provided a quantitative analysis of the binding kinetics and interaction stability. These simulations help elucidate the affinity between Polydatin and ClpP, supporting the hypothesis that polydatin directly modulates ClpP activity to attenuate *S. aureus* virulence. Such simulations offer a deeper understanding of the molecular mechanisms governing the interaction, which is crucial for designing future therapeutic strategies.

In conclusion, our study highlights the potential of polydatin as a therapeutic agent that targets ClpP and modulates *S. aureus* virulence without relying on traditional antibiotics. By interfering with key virulence determinants, polydatin offers a novel approach for combating bacterial infections. This research paves the way for further exploration of natural compounds as modulators of bacterial virulence, providing a much-needed alternative to conventional antibiotic therapies in the fight against multidrug-resistant pathogens.

## Data Availability

The raw data supporting the conclusions of this article will be made available by the authors, without undue reservation.
